# A Review of the Fabrication of Pinhole-Free Thin Films Based on Electrodeposition Technology: Theory, Methods and Progress

**DOI:** 10.3390/molecules29235615

**Published:** 2024-11-27

**Authors:** Zike Gao, Yuze Jiang, Yao Meng, Minshu Du, Feng Liu

**Affiliations:** 1 School of Materials Science and Engineering, Northwestern Polytechnical University, Xi’an 710072, China; gaozike2001@163.com (Z.G.);; 2 Queen Mary University of London Engineering School, Northwestern Polytechnical University, Xi’an 710072, China; 3 Shaanxi Huaqin New Energy Technology Co., Ltd., Xi’an 710119, China; 4 Analytical & Testing Center, Northwestern Polytechnical University, Xi’an 710072, China

**Keywords:** thin films, electrodeposition, pinhole-free, defects, nucleation and growth, electrode

## Abstract

Pinhole defects in thin films can significantly degrade their physical and chemical properties and act as sites for electrochemical corrosion. Therefore, the development of methods for the preparation of pinhole-free films is crucial. Electrodeposition, recognised for its efficiency and cost-effectiveness, shows great potential for applications in electrochemistry, biosensors, solar cells and electronic device fabrication. This review aims to elucidate the role of nucleation and growth models in understanding and optimising the electrodeposition process. Key parameters, such as crystal structure, orientation, surface morphology and defect control, are highlighted. In addition, the causes of pinhole defects, the effects of impurities and the potential and electrolyte composition on the deposited films are discussed. In particular, methods for minimising pinhole defects and two exemplary cases for a compact layer in relatively large-scale perovskite solar cells and nano-scale ultramicroelectrodes are discussed, exploring the influence of surface morphology, thickness and fabrication size under current common film preparation experiments. Finally, the critical aspects of controlled preparation, theoretical and technological advances, and the ongoing challenges in the field are provided.

## 1. Introduction

Pinhole-free thin films are defined as thin films devoid of sharp, round or irregularly shaped depressions. These films are crucial in applications such as electronics, solar cells and biomedical devices. For instance, one of the key challenges in scaling up perovskite solar cells is producing pinhole-free and uniform perovskite and hole transport layers over large areas with high reproducibility [1,2]. Additionally, different scales of composite electrodes are used for catalytic [3,4] or electrochemical process research, and researchers have focused on preparing a pinhole-free electrode surface passivation layer to effectively functionalise the surface properties of the electrodes [5]. Currently, LPCVD (Low-Pressure Chemical Vapour Deposition) and PECVD (Plasma-Enhanced Chemical Vapour Deposition) are the main industrial methods, which are suitable for large area coverage but cannot guarantee high accuracy. Generally, pinhole defects on film surfaces negatively affect the physical and chemical properties of the film and can even act as sites for electrochemical corrosion. Therefore, it is crucial to explore methods for reducing pinhole defects in thin film fabrication. Over the past few decades, atomic layer deposition (ALD) technology has gradually matured, enabling precise control and excellent film quality. It has played an important role in improving the efficiency and stability of solar cells and has been widely recognised and applied. However, ALD also has limitations, such as a slow deposition rate and high-temperature requirements, which limit its application. Electrodeposition is a non-vacuum technique where the electroreduction of ions occurs at a specific reduction potential, forming thin films over a conductive substrate. This method is preferred for producing pinhole-free thin films due to its economic and technical advantages, such as easy scalability, low cost and precise control over film thickness and composition.

The fundamental principle of the electrochemical deposition method is the transport of ions from the electrolyte to the surface of the working electrode by the application of an electric field to facilitate a redox reaction. This results in the nucleation and growth of grains on the electrode surface [6,7]. The electrochemical reactions that occur in the solution encompass both chemical alterations on the electrode surface and kinetic processes, including ion migration and diffusion in the solution, as well as adsorption and desorption on the electrode surface [8]. These processes collectively determine the efficacy of electrochemical deposition and the quality of the deposited layer. A comprehensive understanding of nucleation and the growth process in electrodeposition is essential for producing thin films without pinholes. Generally, the nanoparticle growth process on the electrode surface is considered to be consistent with the typical nucleation and growth model, which was created in the 1960s by Bewick et al. [9]. Avrami [10,11,12] proposed an overlap theory to describe the interactions of nucleus growth in multiple regions on an electrode. Subsequent studies consisted of several stages, and the most widely used models are those proposed by Scharifker and Mostany (SM) [13,14] and Scharifker–Hills (SH) [15,16]. Later, Sluyters-Rehbach, Wijenberg, Bosco and Sluyters (SRWBS) [17] proposed a modification of the SM model by adding overlap into consideration. Heerman and Tarallo (HT) [18] further improved the SRWBS model by introducing the thickness of the uniform diffusion layer into the overlap case. In addition, there is a solution proposed by Mirkin and Nilov (MN) [19], which could be used to calculate the current response with an integral equation. Theoretical models and mathematical analysis of particle dynamics will be reviewed in Section 2.

**Figure 1:**
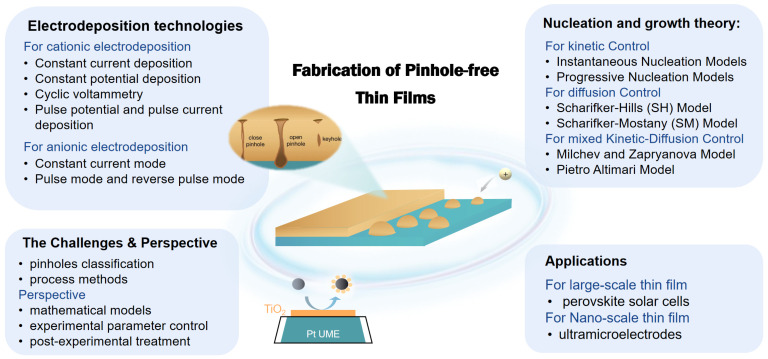
Main content structure diagram of the article.

Though pinhole defects are quite common in films, there is no general discussion of their forming mechanisms so far. Instead, only several articles have pointed out the possible causes of pinholes limited to the specific procedures [20,21,22] or their specific composition [23,24,25,26,27]. This review aims to firstly elucidate the role of nucleation and growth models in understanding and optimising the electrodeposition process in Section 2. The main causes of pinhole defects and the corresponding solutions, including crystallographic methods, for inhibiting the generation of pinholes and the methods for physically plugging pinhole defects are then described in Section 3, which also includes two exemplary cases for a compact layer in large-scale perovskite solar cells and nano-scale ultramicroelectrodes. A summary and outlook are in the last section. The main structure of the article’s content is shown in Figure 1. It is expected to guide the design and preparation of thin films by electrodeposition with less or no pinholes and could also give enlightenment to pinhole controls in other film growth methods, such as CVD (chemical vapour deposition), PVD (physical vapour deposition), the Sol–gel method, etc.

## 2. Theoretical Models and Mathematical Analysis of Particle Dynamics in Electrodeposition Technology

### 2.1. General Description of Electrodeposition Nucleation and Growth Theory

The theoretical description of electrochemical deposition requires consideration of the processes of nucleation, growth and overlapping. To begin with, current is applied to the equilibrium electrode to provide a driving force for nucleation, and the particle–solution interface undergoes a charge transfer reaction, converting diffused ions in the solution to diffused atoms. Next, atoms nucleate at the substrate surface and act as active sites, thus lower energy is required for the subsequent reduction of ions, and then atoms gradually grow to cover the electrode surface and form a thin layer of film.

**Figure 2:**
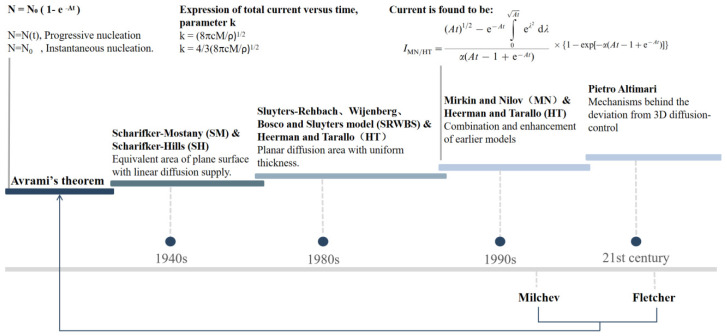
Development of the representative models of nucleation and growth theory.

A series of numerical simulation models have been proposed to interpret the experimental electrodeposition data, the timeline of which is shown in Figure 2. The size of electrodeposited particles is influenced by the rate of early-stage nucleation and the number density of active sites produced. A widely accepted kinetic model is currently used to describe nucleation [9,10], where the time-dependent nucleation density N(t) is:

(1) $$N\left(t\right)=N_{0}\;\times \;(1\;-\;\exp(-A*t))$$

where A and t are the nucleation rate and time, respectively, and N_0_ is the number density of nucleation sites. The overlap of diffusion fields is presented by Avrami’s theorem [10,11,12]:

(2) $$\theta =1-e^{-\theta_{ex}}$$

where θ is the nuclei area, and θ_ex_ is the extended area.

**Figure 3:**
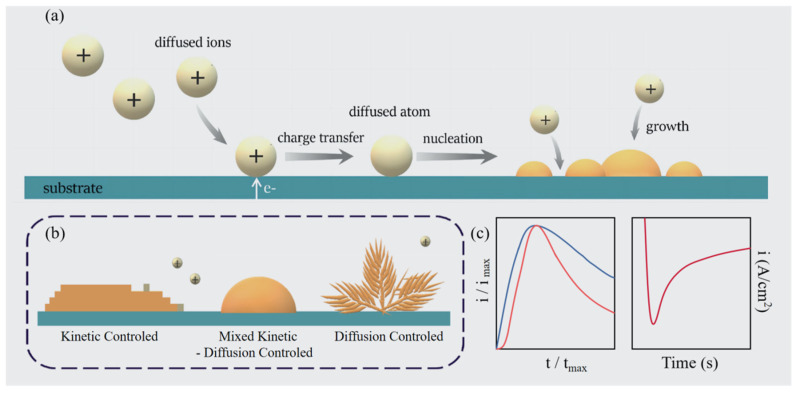
(**a**) The schematic nucleation and growth processes occurring at the electrolyte–electrode interface. (**b**) Classification of the basic shapes of the new phases formed in different growth modes. (**c**) Normalised current versus time curves, in which the blue line represents nucleation in instantaneous process, and the red line represents nucleation in progressive process (**left**); time-dependent current under diffusion control during electrodeposition (**right**).

Over the last few decades, diffusion-controlled growth has been extensively studied based on the formation and growth of isolated three-dimensional hemispherical diffusion zones at static potentials. The models proposed by Scharifker–Hills (SH) [15,16], Scharifker–Mostany (SM) [13,14,28], and Mirkin and Nilov (MN) [19] are able to derive the compact analytical expressions for the dependence of the current transient on nucleation rate A, density of nucleation N_0_ and the diffusion coefficients D. Furthermore, there have been additional models suggested to enhance the accuracy of predicting current transients [29]. The rapid fall of cathodic current during the initial stage of electrodeposition (Figure 3c) has been observed in many systems [30,31], but it is difficult to establish an explanation of the “current–time” relationship. Milchev and Zapryanova [32,33] first proposed the hybrid kinetic–diffusion control model considering the electrochemical behaviour of the multi-step reduction of copper. They argued that a suitable way to explain transient current variations in electrochemical processes is to interpret them as a combination of charge transfer and mass transfer linear diffusion kinetics at planar electrodes. They used asymptotic nucleation and growth theory to take into account charge transfer and diffusion limitations, which can reproduce several experimental results efficiently [34,35,36]. Pietro Altimari et al. [37] built on this foundation with an attempt to address the models’ inability to reproduce planar diffusion transitions by introducing the overlapping of diffusion boundary layers. They proposed an approximate analytical expression for the extended model to calculate the transient currents under the control of mixed kinetic diffusion based on the SM model. In the Pietro Altimari model, the two dimensionless rate constants for growth and nucleation are $\;K_{N}=\frac{A}{DN_{0}}\;\mathrm{and}\;K_{GD\;}$ = $\frac{K_{G}}{D\sqrt{N_{0}}}$, where *K_G_* is the charge transfer characteristic, K_N_ is the nucleation rate constant, and K_GD_ is the ratio of the diffusion time to the charge transfer characteristic, which represents the growth modes.

There are two main mechanisms of nucleation: instantaneous nucleation (K_N_ >> 1) and progressive nucleation (K_N_ << 1) [37]. Instantaneous nucleation involves the gradual development of nuclei on a limited number of active sites, all of which are triggered simultaneously, as shown by the blue line on the left side of Figure 3c. At the beginning of electrodeposition (t = 0), a large number of nuclei are formed in a short period of time, leading to a rapid increase in the rate of electrochemical reaction; thus, the current rises sharply to a high value. As time passes, the current gradually decreases. This is attributed to the fact that as the nuclei grow, the electrode surface is gradually covered, the active sites are reduced, the electrochemical reaction rate decreases, and the current consequently decreases [38]. In contrast, progressive nucleation involves the rapid formation of nuclei on multiple active sites, all of which are activated during electroreduction [15,39]. As illustrated in Figure 3c, the red line on the left side depicts a gradual increase in current during the initial stages of electrodeposition. This phenomenon can be attributed to the formation of nuclei occurring in a step-wise manner, accompanied by a relatively low electrochemical reaction rate at the outset. As the electrode surface is progressively populated with a substantial number of nuclei, the current reaches its maximum value, while the growth processes are distinguished into three categories (Figure 3b), with growth controlled by kinetics when K_GD_ is much smaller than 1, mixed kinetics–diffusion when K_GD_ is close to 1 or diffusion when K_GD_ is much larger than 1 [40]. The shapes of electrodeposited islands are relevant to the different growth modes and will be discussed later.

The growth behaviour of deposited particles during electrodeposition may change. A nucleus can initially grow under the influence of electrodeposition kinetics, but once it reaches a certain size, its growth mechanism may switch to diffusion control; thus, a more complete analysis is necessary [41,42]. The curve on the right side of Figure 3c shows the time-dependent current under diffusion control. At the beginning of electrodeposition, the current rises rapidly. This is due to the high concentration of ions near the electrode surface at the beginning of electrodeposition and the fast diffusion rate, resulting in a rapid increase in the current, which gradually decreases with time. The transition point of the growth mechanism can be determined by analysing the inflection point in the current–time curve. This can be achieved by calculating the second-order derivatives and solving the corresponding time [41,43,44]; we suggest the reader refer to the relevant papers for an in-depth understanding.

### 2.2. Growth Models Under Diffusion Control

Assuming that the only mechanism for electrochemical growth is the direct reduction of ions to the growing nucleus, the equation θ(t) = 1 − exp(−E(t)) can be used to calculate the fractional area θ covered by the diffusion zone. Here, E(t) represents the expectation that diffusion zones cover a representative point on the electrode surface. The derivation of the analytical equation for E(t) is disparate for different models based on different assumptions about nucleation rate and particle radius. The transient current I(t) resulting from the three-dimensional growth of isolated hemispherical particles, irrespective of the growth mode, can be calculated using the concept of a planar diffusion zone introduced by Scharifker and Mostany (SM) [13,14] as

(3) $$I(t)=zFJ_{p}\theta (t)$$

(4) $$J_{p}={\left(\frac{D}{\pi t}\right)}^{\frac{1}{2}}c$$

The mass transfer process uses planar linear diffusion as the equivalent diffusion area by transferring the spherical diffusion to the hemispherical growth centre, J_p_, which represents linear diffusion flux.

In the case of instantaneous nucleation following diffusion-limited growth, the Scharifer–Hill model [15,16] is used to calculate the time-dependent deposition current density. In this scenario, the quantity N(t) remains constant at N_0_, while the surface area A(t) approaches infinity. The model is based on assumptions about the expectation factor E(t) to determine the deposition current density at different times:

(5) $$\theta (t)=1-\exp{\left(-N_{0}D(8\pi^{3}c_{0}V_{m}\right)}^{1/2}t)$$

(6) $$i(t)=\frac{zFc_{0}D^{1/2}}{\pi^{1/2}t^{1/2}}[1-exp(-N_{0}D{\left(8\pi^{3}c_{0}V_{m}\right)}^{1/2}t)]$$

where z is the valence of the metal ions, F is the Faraday constant, D is the diffusion constant, c_0_ is the concentration of ions, and Vm is the molar volume. In this context, it is assumed that the reduction in precursor metal ions is the only Faraday process involved. Therefore, the current transient is directly proportional to t^−1/2^ [9]. The deposition current initially increases as the nucleation area increases and slows down as the nucleus expands. Thus, there will be a current maximum or plateau, and the reach of the current maximum is caused by the overlap of the boundary diffusion layers around the particles. The current decreases as the diffusion length approaches the nucleus spacing, and the nucleus competes for ions [29]. Based on the same theoretical structure, a variety of simulations have been proposed [13,15,16,17,19,28]:

(7) $$t_{max}=\frac{1.2564}{DN_{0}\pi \sqrt{8\pi c_{0}V_{M}}}$$

(8) $$I_{max}=\frac{0.7153zFD^{1/2}c_{0}}{\sqrt{\pi t_{max}}}=1.429zFDc_{0}^{5/4}V_{M}^{1/4}\sqrt{N_{0}}$$

To analyse the deposition transient, compare it with the growth law normalised to the maximum current (I_max_) and the time (t_max_) at which the maximum current is observed. When dealing with instantaneous nucleation:

(9) $$\frac{i^{2}}{I_{max}^{2}}=1.9542\frac{t_{max}}{t}{\left[1-exp(-1.2564\frac{t}{t_{max}})\right]}^{2}$$

However, ion discharges are indeed reduced to atoms accumulating on the electrode surface, and at lower nucleation rates, the accumulation of ions leads to a drop in current transients over a short period of time, as electron and/or ion transfer reactions may occur “before” and/or “simultaneously” with nucleation, resulting in progressive nucleation. In the case of progressive nucleation and the subsequent diffusion-limited growth, the time-dependent deposition current density can be calculated using the equation N(t) = k_n_N_0_t and the following equation:

(10) $$\theta (t)=[1-exp(-\frac{4}{3}K_{n}N_{0}D{\left(2\pi^{3}c_{0}V_{m}\right)}^{1/2}t)]$$

(11) $$i(t)=\frac{zFc_{0}D^{1/2}}{\pi^{1/2}t^{1/2}}[1-exp(-\frac{4}{3}K_{n}N_{0}D{\left(2\pi^{3}c_{0}V_{m}\right)}^{1/2}t^{2})]$$

The current transient for progressive nucleation can be normalised as follows:

(12) $$\frac{i^{2}}{I_{max}^{2}}=1.2254\frac{t_{max}}{t}{\left[1-exp(-2.3367\frac{t^{2}}{t_{max}^{2}})\right]}^{2}$$

### 2.3. Growth Models Under Mixed Kinetic–Diffusion Control

The process of electrochemical nucleation and growth can be quantified by multiplying the planar diffusion flux on the electrode surface with the generated current, $J_{P}\pi R_{P}^{2}=J_{D}2\pi R_{D}^{2}$. This equation also allows for the derivation of the radius of the diffusion zone. In this equation, R_D_ represents the radius of an isolated hemispherical particle, while J_P_ and J_D_ represent the planar and spherical diffusion fluxes, respectively, both operating under mixed kinetic–diffusion control.

The extended current density i_ex_ (t) improves current transient prediction by replacing zFJ_P_ in Equation (3) with i_ex_ (t)/E(t):

(13) $$i_{ex}\left(t\right)=zF\int_{0}^{t}2\pi R_{D}^{2}\left(t-u\right)J_{D}\left(t-u\right)\frac{dN}{du}du$$

(14) $$E\left(\tau \right)=\pi {\bar{R}}_{P}^{2}\left(\tau \right)-2\pi exp\left(-K_{N}\tau \right)\int_{0}^{\tau }\left(1+K_{N}u\right){\bar{R}}_{P}\left(u\right)\frac{d{\bar{R}}_{P}\left(u\right)}{du}du$$

The expression for the current density can be obtained by combining Equations (13) and (14), which is given by [37]

(15) $${\bar{I}}_{V}\left(\tau \right)={\bar{J}}_{P}\left[1-exp\left(-\pi {\bar{R}}_{P}^{2}\left(\tau \right)+2\pi exp\left(-K_{N}\tau \right)G\left(\tau \right)\right)\right]$$

Altimari and Pagnanelli employ expressions to compute the expectation factor (Equation (15)), and the derived analytical solution is applicable in the limit of transient and asymptotic nucleation, which provides a satisfactory approximation of the current transient for any value of K_N_.

### 2.4. Growth Models Deviated from 3D Diffusion Zone Control

The current model is largely capable of describing the relationship between the current transient and nucleation and growth behaviour during a single Faraday reaction. However, for the 3D diffuse zone growth mode, where the contribution of diffusion from the surface of the atoms is neglected in the original assumption, the direct reduction of the metal ions into the growing nuclei is considered to be the only mechanism for electrochemical growth. In fact, during the early stage of electrodeposition, metal ions may discharge into arbitrary aggregates of atoms on the electrode surface, forming nanoclusters that migrate randomly across the surface until the clusters are incorporated into the nuclei or re-crystallisation occurs, allowing growth by the direct attachment of metal ions [45,46,47]. Arbib [48] found a deviation from the first-order nucleation through observation of the current transients in the 2D nucleation and growth process to the first-order nucleation kinetic theory. The formation of 3D clusters on top of the 2D phase is consistent with the SM growth mode, and the effect of two-dimensional growth on the current is thought to be related to a reduced rate of aggregation of the clusters, and a correction to N(t) is also proposed. Mamme et al. [47] pointed out that the surface mobility of nanoclusters might have a more significant effect on the number and size distribution of clusters than the applied overpotential. The behaviour of clusters has an important influence on the growth mode of the film, but the effects of cluster nucleation, aggregation and migration on the current generation are currently difficult to quantify.

In the case of 2D nucleation, Equations (16) and (17) describe the behaviour of transient and progressive growth given by Ref. [49].

(16) $${\left(\frac{i}{I_{max}}\right)}_{Inst}=\frac{t}{t_{max}}exp\left(-\frac{1}{2}\left(\frac{t^{2}-t_{max}^{2}}{t_{max}^{2}}\right)\right)$$

(17) $${\left(\frac{i}{I_{max}}\right)}_{Prog}={\left(\frac{t}{t_{max}}\right)}^{2}exp\left(-\frac{2}{3}\left(\frac{t^{3}-t_{max}^{3}}{t_{max}^{3}}\right)\right)$$

where I_max_ and t_max_ are the maximum current and time to reach the maximum current, respectively. Similarly, Equations (9) and (12) describe the 3D growth mechanism as proposed by Sharifker and Hills [15] above. Mathematical models based on the direct attachment inhibition of electrolyte anions [48,50,51,52] and direct metal–ion discharge to the substrate have also been suggested to help identify a well-defined system by the polymerisation and recrystallisation of nanoclusters or the adsorption of chemical compounds and the precipitation of resistive compounds [50], rather than coming from overlapping diffusion regions and interactions between nuclei, as mentioned in [53].

In addition, 2D-3D and 3D-2D transitions have been observed in many experiments [51,54], which can enlighten the design of thin films in terms of thickness and particle size, and remind experimental designers to consider not only the overpotential, reactive species transport and electrochemical kinetics but also adsorbed atoms that are generally neglected in electrochemical nucleation and growth models.

### 2.5. Particle Dynamics Theory Models and Their Specific Applications in Electrodeposition

In the field of electrodeposition, anodic techniques have been dominant, employing a variety of methods, including constant current [55], constant potential [56,57,58], pulsed potential and pulsed current deposition [59,60,61,62]. In contrast, cathodic deposition methods, although less reported, highlight an area of research with significant potential. These cathodic deposition techniques, including constant current and pulsed and reverse-pulsed modes [63], are capable of achieving deposition on different metal substrates without inducing anodic oxidation of the substrate and offer the possibility of co-depositing other materials with the promise of enhancing their electrochemical properties.

In the electrodeposition process, direct current (DC) potentiostatic technology provides a stable overpotential to overcome the resistance of electrochemical polarisation, concentration polarisation and resistance polarisation by applying a constant current or potential so that metal ions continue to reduce and form deposits on the electrode surface. In pulsed electrodeposition, a current or potential is applied in the form of pulses, during which a higher current density is applied to provide a large overpotential to accelerate the reduction and nucleation process of metal ions. The pulse intermission period allows reactant diffusion and electrolyte recovery, thereby reducing concentration polarisation and improving the quality and uniformity of the sediment.

The Nernst equation shows that the deposition process is driven by the overpotential, denoted as η, calculated as the difference between the applied potential ∆E and the concentration overpotentials ηC and ohmic overpotentials η_Ω_ [43,44]

(18) $$\eta \;=\;∆E\;-\;\eta_{c}\;-\;\eta_{\Omega }$$

The overpotential, as an experimentally measured parameter, is thought to be able to establish a link with the pattern of nucleation and growth of nuclei, thus enabling control of the size and density of particles of thin films. For most systems of technical interest, if the overpotential is large, deposition is at least partially controlled by ion diffusion in the bulk solution. For small nucleation potentials, island growth is in a kinetic state, making it possible to design the shape of the deposited nuclei [64]. In order to effectively control nucleation and growth processes, researchers have explored techniques, such as the implementation of double-step potential [65,66]. These techniques have been developed to control the shape as well as the arrangement of the deposited particles by first applying a high overpotential to form a considerable number of nuclei and, secondly, by applying a lower overpotential to control the reduction mechanism of the metal ions.

The numerical simulations presented by Isaev et al. [67] illustrate the fundamental distinction between potentiostatic current transients in kinetically and diffusion-controlled growth processes. At the beginning of the deposition, the overpotential η increases instantaneously. For kinetic-controlled growth, the overpotential decreases to a stable plateau level, while for diffusion-controlled growth, a dynamic decrease in the overpotential (η) is observed. The sudden increase in η seems to be a commonly occurring abnormal evolution. The variation in electrode potential can affect the distribution of reaction rates for different reactions, such as multi-step reactions or reactions in the forward and reverse directions.

**Figure 4:**
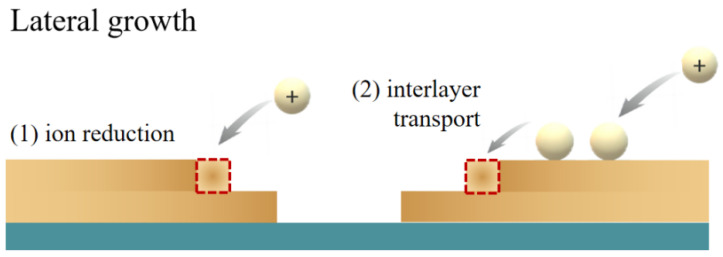
Diagram illustrating the lateral growth process of islands on a foreign substrate, involving two mechanisms: (1) direct attachment through the reduction of metal ions and (2) interlayer transport. The red dotted box indicates where the ion is adsorbed.

According to atom-to-atom and atom-to-substrate bonding forces, electrodeposited thin films are mainly classified into three growth modes: the Frank–van der Merwe layered growth mode (FVDM mode), the Volmer–Weber island growth mode (V-W mode) and the Stranski–Krastanov hybrid growth mode (S-K mode). The link between overpotentials and growth modes has been explored. Under kinetic control at low overpotentials, the deposited atoms grow, and the shape of the layer is determined by the energies of the different surfaces. Deposited atoms under mixed kinetic/diffusion control usually lead to the formation of hemispherical shapes, while under diffusion control at higher overpotentials, aggregates or dendrites form. Based on this, it is desired to reduce pinhole defects on the surface through island morphology control and the enhanced lateral growth of islands. Direct attachment of ions to atoms leads to vertical growth, while indirect attachment leads to the attachment of peri-island adsorbates and contributes to lateral growth, as shown in Figure 4. Methods to reduce overpotential, such as electrodeposition on the self-assembled monolayer [68], have also been widely reported for the construction of dense films.

In pulse and reverse-pulse electrodeposition, the factors affecting nucleation and growth mainly include double-layer charging, mass transfer process, pulse current characteristics, duty cycle and frequency, current distribution, power consumption, additive use and special techniques in alloy deposition [69,70]. In particular, the mass transfer process determines the supply of metal ions and affects the number of nucleation and grain refinement; a flatter surface morphology can be achieved through the synergistic effect of duty cycle and frequency [71]. The pulse frequency and duty cycle are controlled by the on-time (t_ON_) and off-time (t_OFF_) of the current. During t_ON_, both electromigration (EMD) and diffusion (MSD) are active, while during t_OFF_, only diffusion is active. As the diffusion layer does not grow excessively at high frequencies, higher frequencies result in smaller grain sizes, leading to a more orderly geometric structure of the particles. Changes in the duty cycle directly affect the lengths of t_ON_ and t_OFF_, thereby influencing the relative action times of electromigration and diffusion [72]. Therefore, increasing the frequency and reducing the duty cycle can significantly suppress 3D growth on the thin film surface when the current and external circuit are constant.

Pulse and reverse-pulse electroplating technology has shown unique advantages in the deposition of various metals and alloys. For metal alloys, composites and semiconductor thin films, this technology enables the precise control of the performance and physical and chemical properties, promoting grain refinement and corrosion resistance. These technological advances provide a broad space for the development and application of new materials.

## 3. Formation Causes of Pinholes and Routes of Pinhole-Free Film Preparation

**Figure 5:**
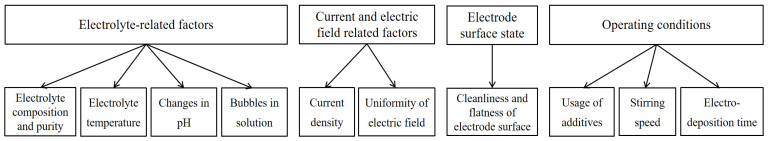
Main causes of pinhole defects and the corresponding solutions.

The formation of film defects during electrochemical deposition involves several levels of influencing factors. Macroscopically, these factors can be grouped into four broad categories: electrolyte-related factors, current and electric field-related factors, electrode surface states and operating conditions. These four categories of factors shown in Figure 5 provide a broad classification framework to help identify and control potential sources of defects.

In order to gain a deeper understanding of the mechanisms of defect formation, we have refined these factors into seven categories: surface and interfacial phenomena, material properties, electric field and electrochemical conditions, thermodynamic and kinetic factors, stress and mechanical effects, impurity–defect interactions and the dynamics of the deposition process. These factors do not exist in isolation but work together to influence film quality through complex interactions. For example, the composition of the electrolyte influences the electrochemical conditions, while the strength of the electric field affects surface and interfacial phenomena. Thus, a comprehensive understanding of the formation mechanisms of thin film defects requires a combination of macroscopic classifications and the interaction of specific factors. In the next section, we will explore these seven factors in detail, revealing their specific roles and inter-relationships in the formation of thin film defects.

### 3.1. Formation Causes of Pinhole Defects

**Figure 6:**
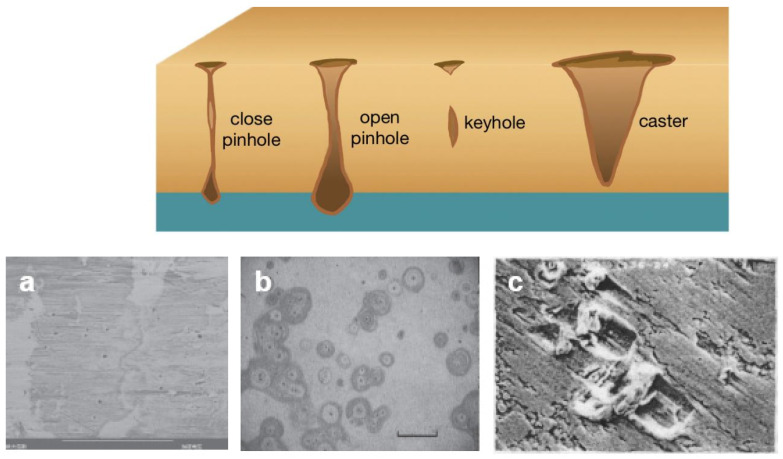
Schematic diagram of different shapes of hole-like defects in the electrodeposited films (upper). (**a**) The presence of dust contamination has resulted in high porosity. (**b**) The corrosion stains on a thin gold-plated surface. (**c**) Pits caused by contact with hard particles [74]. (Reproduced with permission from the source [67]. Copyright (2014) Taylor and Francis Group LLC. (Books) US; CRC Press.).

Defects in electrodeposited films are exposed primarily through micron-sized pores and defects in thin-plated layers, leading to corrosion of the substrate [73]. Complex chemical reactions produce corrosion products that form corrosion build-up and enlarge the defects [74]. Hole-like defects (e.g., closed pinholes, open pinholes, keyholes and casting defects, as shown in Figure 6) produced during electrodeposition are very common, and the formation of these defects can be attributed to a variety of factors.

**Figure 7:**
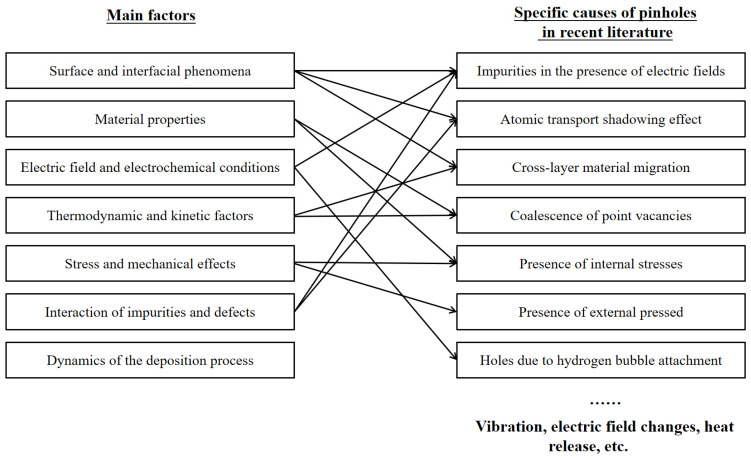
Causation of pinhole by interaction of factors.

Specifically, research in recent years has identified impurities in the presence of electric fields, atomic transport shadowing effects, aggregation of point defects, cross-layer material migration, internal stresses, external pressures and pores formed by hydrogen bubbles as common causes of defect formation (Figure 7). These modes of defect formation are explained specifically in the following section:

(i) Impurities in the presence of electric fields

Defects and impurities on the substrate surface can lead to the nonuniform deposition of ions and the formation of pinhole defects, which enhance the aggregation of particles in the interstitial region under the action of the electric field. The greatly enhanced electric field strength in the interstitial region can be shown by the finite element method (FEM), and the experimental results verify the higher particle density in the interstitial region [74]. The particles move from the region where the pinholes are far away to the high field region between the pinholes and the flat plate. Studies have shown that changes in electric field strength can significantly affect particle movement and deposition. In an electric field, vibrations during opening and closing may dislodge particles adhering to other surfaces, and particles may be attracted to the contact surface by the electric field. Dust deposition is selective, especially on highly charged particles, such as organic materials and titanium dioxide, which have a higher probability of being on the contact surface;

(ii) Atomic transport shadowing effect

Also called Fjord closure, this is a phenomenon where holes are formed and cannot be covered by electrodeposited atoms, deriving from the growth of islands that block the transport of the subsequent atoms into the neighbouring atomic nucleus [24]. Impurities, defects or edge effects on the substrate surface can lead to the nonuniform deposition of ions, where diffusing atoms or diffusing clusters are blocked or preferentially adsorbed during transport, leading to the formation of cavities where the subsequent diffusing atoms or diffusing ions are blocked. For crystalline thin films, pinhole defects can arise inside the crystal and can also be concentrated at grain boundaries [75]. For example, Ji et al. [76] investigated the realisation of ultra-nonlinear selective switches in electrodeposited control films by combining high defect density chalcogenide glass with high mobility silver elements, providing a new direction for selector design;

(iii) Cross-layer material migration

In materials with multi-layer structures, cross-layer migration of atoms or molecules occurs between different layers. Such migration processes are affected by a variety of kinetic factors, including interfacial energy and thermodynamic driving forces, or are induced by redox [77,78]. For example, migration between neighbouring atoms during deposition on a soft metal substrate is strongly correlated with the surface interface energy. A study by L.D. Roelofs et al. [24] revealed how spontaneous doping of deposited atoms drives substrate atoms to migrate towards the surface, which triggers the formation of pinholes [79]. Specifically, two neighbouring atoms initially move in a co-planar on the substrate, a phenomenon known as upward exchange diffusion (UED), i.e., one atom moves down to occupy an empty space in the next layer, while the other occupies the former’s previous position. This jumping motion of atomic layers occurs with the change of surface energy and, in turn, leads to the re-modelling of the surface structure.

On the other hand, the thermally driven material migration and phase transition phenomenon (TDMTP) is manifested in some chemical reactions with low activation energies, like silicide formation [80]. Heat release accompanying the reaction, which leads to the melting of the silicide and the substrate layer, can create pinholes;

(iv) Coalescence of point vacancies

This refers to the phenomenon where the density of vacancies that have not been nucleated increases, causing them to merge and form cavities. Point vacancies are formed when atoms in a crystal structure deviate from their regular lattice positions or fail to occupy them [81,82]. This can occur due to various factors, such as preferential behaviour, thermal energy, reactions or phase transitions, leading to the creation of point defects [83]. The density of defects in a material, including vacancies, antisites and interstitials, as well as their charge states (positive, negative or neutral), is determined by the chemical potentials of the atoms and electrons that make up the material. The energetics of these defects, diffusion paths, migration energies and energy barriers for reactions between these defects can be calculated and predicted by first principles [84,85];

(v) Presence of internal stresses

The presence of internal stresses within the electrodeposited layer is a common phenomenon, and excessive levels of residual stress can significantly affect the performance, reliability and durability of material components and devices [86,87]. Internal stresses are classified into macroscopic internal stresses and microscopic internal stresses. The former is classified as tensile and compressive stresses, both of which may cause pinhole generation. Microscopic internal stress is only limited to the range of grain size. There are many factors that affect the internal stress in a layer, including the electrodeposition conditions, solution system, substrate material, etc. The formation of 2D vacancy islands observed in metal–metal heteroepitaxy [88] is considered beyond the three classical growth modes and is suspected to be driven by tensile stresses;

(vi) Presence of external pressed

External mechanical pressure or stress can also affect the structural integrity of films, leading to the formation of defects [89,90];

(vii) Pits or holes formed by attached hydrogen bubbles

The formation of metallic structures may be accompanied by a hydrogen co-deposition process [91,92]. The two ways in which hydrogen co-deposition triggers the formation of pore structures are as follows: first, hydrogen uptake may take place in the form of H atoms in the base metal, where hydrogen collects in the form of molecular bubbles into the voids or vacancies, leading to hydrogen embrittlement; second, hydrogen bubbles attach to the surface in an adsorbed state, and the deposits around the bubbles continue to deposit until the bubbles are released, leading to the growth of pores [93]. In addition, a decrease in solution pH or an increase in the proton reduction rate coming from an increase in overpotential may lead to an increase in the amount of hydrogen adsorption as well as an enhancement of the effect [50,94,95].

### 3.2. Discussion on Parameter Adjustment in the Electrodeposition Process

#### 3.2.1. The Role of Anions in Influencing Nucleation, Growth and Particle Shape in Electrodeposition

The importance of anions in terms of their influence on the nucleation pattern, growth pattern and particle shape is emphasised in the literature [51]. The strength of the electrolyte has an important effect on the current passing through it. For example, chloride ions are considered to be deposition inhibitors that reduce the overpotential due to their adsorption with various metal atoms [96], and Li [97] recently reported oxygen vacancies on TiO_2_ that help to enhance the adsorption of halide ions. Though the actual effect of chloride ions on surfaces is not yet known, it should be used as an error factor influencing the researcher’s choice of the electrolyte solution.

Anions are able to influence the growth mechanisms of thin films, especially at 2D-3D and 3D-2D transitions. The growth pattern of islands was examined by Eduardo. N. Schulz et al. [51] for Rh on Ag electrodes with different overpotentials on nucleation and growth mechanisms in solutions containing chloride ions. It was reported that the main process at lower deposition potentials was instantaneous nucleation and 2D growth, while at higher overpotentials, a 2D-3D growth mechanism transition occurred. Only 3D growth mechanisms can be observed in sulphate media (higher overpotentials), suggesting that the simultaneous deposition process is significantly dependent on anions present in the electrolyte. The effect of anions on the shape of deposited atoms is also significant. Guo [98] reported the electrochemical growth of copper particles on ruthenium oxide substrates in sulphate and perchlorate solutions, respectively, with dramatically different particle shapes and nucleation rates. A hemispherical and polycrystalline morphology and progressive nucleation with more diffuse atom migration were exhibited in perchlorate. As a result, the two- or three-dimensional growth of thin films can be modulated by the direct attachment inhibition/induction of electrolyte ions [99], and thin films with single- or bilayer atomic heights can be designed by modulating the balance of cluster nucleation and aggregation. Reference [47] provides some conclusions on the number, size and mobility of the clusters that can be used as a reference.

#### 3.2.2. Impact of Point Defects and Surface Diffusion in Electrodeposition

According to the point defect model (PDM) [23] in oxide, oxygen ion vacancies form and annihilate following atomic migration, which then evolve into point defects, and the migration and annihilation of cation vacancies/anion vacancies evolve into a point source [100]. In the case of point defect formation on the surface of multinary chalcogenide materials, for example, the conversion of the sulphur group of compounds to cationic oxides on exposure to air drives the preferential removal of the cation and thus the formation of the anionic vacancy. For sulphide compounds where zone edges exist, differences in the thermodynamic dominance of the electrochemical potential of the relevant substances at the phase boundary may exacerbate vacancy formation [83]. The addition of large-scale ions to occupy some of the ionic vacancy formation sites, creating a “site-blocking” effect, is thought to be a way to reduce the number of pitting sources.

In the early growth of metal–metal heteroepitaxy [24], point vacancies may form because the bonds between the deposited atoms and the substrate atoms are stronger than the bonds between the substrate atoms. The stronger bonds first lead to a decrease in the density of the epitaxial substrate atoms, which, in turn, drives an increase in vacancy density through the law of mass action. Secondly, the addition of deposited atoms decelerates the movement of vacancies through the platform by immobilising adjacent substrate atoms, and this slowing down further increases the vacancy density.

The formation of point vacancies is related to the binding energy between atoms, the activation energy for surface diffusion and the overpotential ղ in electrodeposition [101]. The lower the binding energy between the deposit and the substrate, the higher the surface tension and the stronger the surface diffusion. The diffusion of surface atoms is a combination of creation and annihilation events, where atoms and small clusters move randomly along the electrode surface and then aggregate with each other in order to minimise the excess of surface energy. Surface diffusion decreases with increasing cluster size. The surface diffusion coefficient D for one of these clusters can be defined as:

(19) $$D=D_{0}\cdot N_{adatoms}^{-\alpha }$$

where D_0_ is the surface diffusion coefficient of an atom, N_adatom_ is the number of atoms forming clusters, and α indicates the relative mobility factor for small and large clusters (α ≥ 0). The interaction between the deposited metal and the substrate can be enhanced and the vacancy migration of surface atoms reduced by adjusting the surface migration rate, e.g., using underpotential deposition (UPD).

#### 3.2.3. Strategies for Pinhole Mitigation in Thin Films

The construction of fully covered films sometimes needs to be achieved at the expense of film thickness, and it has also been argued that in atomically thick films, the optimisation of deposited parameters alone cannot completely eliminate pinholes, as the formation of the film is inevitably affected by a number of dynamic factors, such as the surface migration of atoms, adsorption, recombination, etc. Thus, additional methods to plug pinholes in films are proposed.

I. Self-passivation of thin films. Hyun S. Ahn and Allen J. Bard [102] proposed an interesting way of constructing pinhole-free films through self-passivation using TiO_2_ to construct charge-transferring tunnelling barriers on a n-Si substrate. After initial dehydration of the electrodeposited film, pinholes and uncovered regions were observed. Then, the Pt/TiO_2_/n-Si electrode was immersed in oxygen-saturated water for a period of time, and a SiO_2_ passivation layer was finally grown to fill the pinholes of the film with a current shielding rate of 99%;
II. Defect repair films. A number of organics can be used to repair defective dielectrics. For example, polystyrene oxide (PPO) is an electrically insulating solid polymer film that can be prepared by the electropolymerisation of phenol on a conductive surface [103]. Macech et al. [104] used PPO with a thickness of about 6 nm to plug pinholes in a silica film on a gold substrate. PPO can also be used directly as a blocking layer [105] for reducing the self-discharge of supercapacitors [68]. Hicham Amegroud et al. [106] deposited a polyaniline (PANI) conductive polymer as a protective layer using electrostatic polymerisation with varying current densities and achieved good corrosion resistance.

### 3.3. Discussion on Typical Cases of Pinhole-Free Film Electrodeposition

I. A case of a perovskite solar cell compact layer

**Figure 8:**
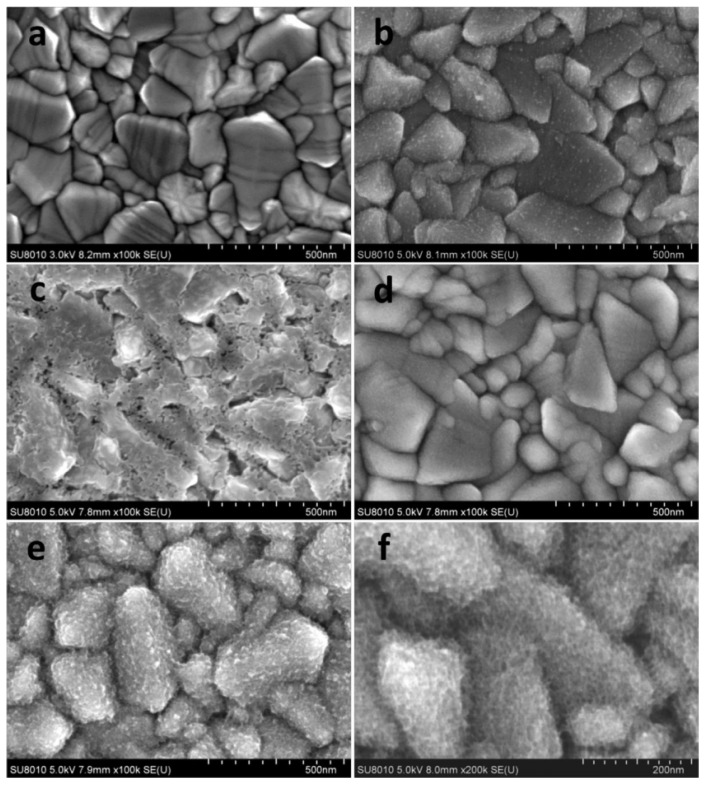
SEM images of (**a**) bare FTO and (**b**) DC-BL, (**c**) SC-BL, (**d**) SP-BL and (**e**) ED-BL on FTO at 100k magnification and (**f**) ED-BL on FTO at 200k magnification [121]. (Reproduced with permission from Ref. [113]. Copyright (2017) John Wiley & Sons books.).

In perovskite solar cells (PSCs), high-quality blocking layers (BLs) are used to prevent the charge carriers from directly contacting the conducting substrate, which is crucial for cell efficiency. Titanium dioxide is the most commonly used BL material and is generally fabricated by spin coating (SC) [107,108,109,110], spray pyrolysis (SP) [111,112,113], atomic layer deposition (ALD) [114,115,116], chemical deposition [117], thermal oxidation [118,119], etc. Spin coating and spray pyrolysis, as two representative methods, have the advantage of simplicity, economy and scalability, but both suffer from sensitivity to process parameters, incomplete coverage, possible pinholes, resistive losses and lower thermal stability and blocking effect. Hong et al. [120] fabricated low-cost and high-density TiO_2_ films by dip-coating, but the dip-coating (DC) method is constrained by film thickness and surface morphology. Electrodeposition (ED) is an economical, straightforward and scalable technique that allows for the production of even and seamless surface coatings without the necessity of high-temperature processing or vacuum conditions [81,121,122,123,124]. The BLs prepared by electrodeposition also exhibit smoother and more uniform morphology, as shown in Figure 8. More importantly, the rate of electrodeposition is an order of magnitude higher than that of atomic layer deposition, which is advantageous when considering the large-scale production of solar cells [81]. A detailed comparison of various methods for pinhole-free blocking layer preparation in PSCs is listed in Table 1.

Also, a variety of modifications ranging from inorganic and organic materials to nanocarbon have been reported to modify the electronic properties and reduce the surface defects of BLs [125]. Anuratha et al. [126] reported the use of surfactants to enhance the porousness of TiO_2_ through an electrodeposition process. A constant current deposition method was used to fabricate bifunctional TiO_2_ thin films, with Brij 58 as a soft template [12]. This technique addresses the issue of pinholes in the blocking layer by modifying them and simultaneously serves as a porous scaffold layer capable of accommodating a significant amount of chalcogenide. The use of Brij 58 as a soft template in the electrodeposition method presents a promising approach for creating an efficient scaffold-blocking layer in perovskite solar cell (PSC) applications.

**Table 1:** Comparison of the methods for TiO_2_ compact layer preparation in PSCs.

Methods	Electron Deposition (ED) [81,121]	Dip Coating (DC) [120]	Spin-Coating (SC) [120]	Spray Pyrolysis (SP) [120]	Atom Layer Deposition (ALD) [127]	Thermal Oxidation Method [128,129]
Applicable device size	Small size, micron to sub-micron	Medium size, micron to millimetre	Small size, micron to sub-micron	Medium size, sub-micron to millimetre	Small size, nanometre to sub-micron	Large size, millimetre to centimetre
Thickness of the films produced	~35 nm	~55 nm	75–127 nm	50–100 nm	2–50 nm	300–350 nm
Preparation speed	Relatively short	Relatively long	Short	Short	Relatively long	Long
Film production characteristics	High controllability and deposition efficiency	Simple and economical but usually requires multiple dips to achieve thickness	Cost-effective for small-scale production or laboratory studies	Enables large and uniform film preparation	Very high film uniformity and controlled thickness	Not suitable for smaller device sizes, requires more time and higher equipment costs
Batch preparation in industry	Y	N	N	Y	Y	N

II. A case of pinhole-free film electrodeposition on ultramicroelectrodes

Pinhole-free thin film deposition on ultramicroelectrodes (UMEs) has important applications in the field of electrochemical analysis and single nanoparticle collision electrochemistry [130,131]. It is designed for tunnelling UMEs as a novel nanometre-size electrode, i.e., the pinhole-free thin blocking layer deposited on the conducting Pt UME blocks electron transfer to the solution species, and the layer should be thin enough to enable tunnelling to the subsequent contacted metal nanoparticles (NPs) [5]. Similarly, Wei Ma et al. [68] prepared pinhole-free monolayers on a Au UME surface by combining octadecanethiol self-assembled monolayers with electrodeposited tyramine polymers, the key to which is the reduced nucleation overpotential and the higher substrate adhesion during electrodeposition. The prepared tunnelling UME was used for core-shell metallic nanoparticles in situ electrodeposition and instantaneous electrocatalytic measurement. It should be mentioned that for deposition on UMEs or microelectrodes, fewer active sites and higher current densities should be expected due to smaller substrate areas, and the electrodeposition exhibits the following main characteristics: (1) Mononuclear nuclei may form on electrodes with dimensions smaller than the diffusion region, and there are techniques to scale up the dimensions of individually nucleated microelectrodes to several hundred nanometres [41,132]. (2) The deposition is controlled by diffusion at high overpotentials due to larger current densities [44]. (3) Microelectrodes are more subject to edge effects [133].

**Figure 9:**
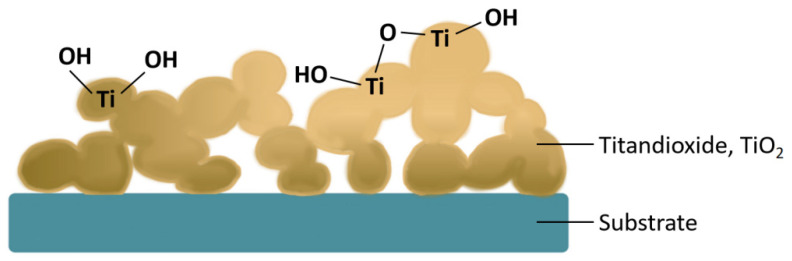
Schematic of the surface morphology of the samples electrodeposited in the TiO_2_ layer.

We reproduced the pinhole-free TiO_2_ thin film electrodeposition on a 25 μm Pt UME by improving the procedures reported by Jiyeon Kim et al. [5]. Converting the TiCl_3_ solution into a TiO_2_ film on the anode involves a two-step reaction, and Figure 9 is a schematic of the surface. First, TiCl_3_ undergoes hydrolysis, resulting in the formation of Ti(III)OH^2+^ and a proton (Equation (20)). Subsequently, Ti(III)OH^2+^ undergoes oxidation, resulting in the formation of polymeric Ti(IV) hydroxide, also known as TiOx. Ti(IV) hydroxide oxidises further to form TiO_2_ (Equation (20)) under ambient conditions [134]. The Pt UME was pretreated and immersed in an electrolyte containing 1 mM ferrocenemethanol (FcMeOH) and 300 mM KNO_3_ to obtain the cyclic voltametric (CV) curves. The UME was then deposited through electrodeposition in an electrolyte of 50 nM TiCl_3_ and 100 mM KNO_3_ with the potential set at 20 mV above the open circuit potential.

After each electrodeposition, the electrode was left to dry for 24 h or exposed to an infrared lamp for 1 h to ensure complete conversion of TiOH^2+^ to TiO_2_.

(20) $${Ti}^{3+}(aq)+H_{2}O\overset{fast}{↔}{TiOH}^{2+}+H^{+}$$

(21) $${TiOH}^{2+}-e^{-}\overset{slow}{\to }Ti(\mathrm{IV})\;polymers\;\to {TiO}_{2}$$

At the start of the deposition process, the interface between the exposed electrode and the electrolyte is in ohmic contact. However, as the deposition process progresses, the electrode surface becomes covered with polymerised titanium(Ⅳ) hydroxide. Due to its semiconducting nature, the electrode interface becomes a Schottky contact, which creates an energy barrier to the reverse reaction, resulting in a decrease in the surface overpotential. This, in turn, affects the rate distribution of the first-step reaction, shifting the reaction towards the forward direction and further promoting the formation of the deposition layer [135,136,137].

**Figure 10:**
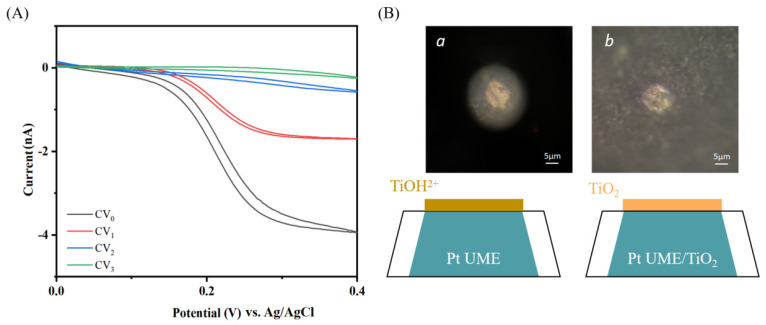
The reproduced electrodeposition of pinhole-free TiO_2_ thin films on Pt UME by authors. (**A**) Cyclic voltammetry curves with increasing deposition rounds (from CV_0_ to CV_3_) in 1 mM FcMeOH solution. (**B**) SEM images of the TiO_2_ thin film on the Pt UME surface (**a**) after electrodeposition and (**b**) after electrodeposition and oxidation.

The UME was then subjected to a CV curve test to monitor the blocking effect of the TiO_2_ film by comparing it with the bare Pt UME. As shown in Figure 10A, deviating from the typical Z shape, the complete blocking of electron transfer to the redox species in the solution was obtained after about 3–5 rounds of electrodeposition and the drying process, which also indicates the pinhole-free state of the electrodeposited TiO_2_ thin films on the UME. When the first step (Equation (20)) was completed during electrodeposition, as shown in Figure 10B(a), it can be observed that a thin film formed on the electrode surface. The film consisted of titanium hydroxide, which was relatively thin and evenly distributed. The film exhibited a subtle, colourful sheen. However, after the electrode completed deposition and underwent natural oxidation, the surface of the TiO_2_ deposition layer exhibited a distinctly different morphology. The boundaries of the film appear blurry, resembling a coverage of moss-like deposits. The observed morphology, similar to that observed by Su et al. [121], can be attributed to the fact that titanium (IV) polymers are not effective electronic conductors. In contrast, the moss-like structure provides more contact sites for the TiO_2_ framework, reducing contact resistance and facilitating efficient electron tunnelling transfer [134], which can correspondingly improve efficiency and performance in device applications.

In summary, achieving pinhole-free thin film deposition on ultramicroelectrodes (UMEs) is essential for reliable electrochemical applications. To reduce pinholes in thin films, precise control of the deposition conditions is critical. This involves optimising parameters such as nucleation overpotential, substrate adhesion and cyclic voltametric settings. For example, a process involving the conversion of TiCl_3_ to TiO_2_ on Pt UMEs uses controlled hydrolysis and oxidation followed by drying cycles to ensure complete film formation. Such meticulous control helps to produce uniform, pinhole-free films, improving the performance and reliability of UMEs.

## 4. Summary and Outlook

Electrodeposition methods provide a means of forming high-density deposited layers of controlled thickness on a substrate, which is efficient and economical in laboratory preparation and industrial production, and is widely used in the fields of electrochemistry, biosensors, solar cells and electronic device preparation. During the electrodeposition process, different phase structures ranging from 1D to 3D can be formed through fine and real-time experimental control [138], such as the selection of appropriate deposition modes, applied overpotentials, electrolytes and additives.

Since the last century, nucleation and growth theories have been developed through a combination of experimental observations and theoretical analysis, and analytical expressions have been derived describing the relationship between the current transient and the nucleation rate, the nucleation density and the diffusion coefficient [139]. These theories are important in understanding and guiding the actual electrodeposition process. The type of kinetic control, diffusion control or hybrid kinetic–diffusion control determines the growth mode of the crystals on the surface and the different aggregation morphologies. On this basis, it is desirable to increase the mobility of the atoms or ions by increasing the temperature and the deposition rate or lowering the overpotential moderately to enhance the lateral growth of the islands and reduce the generation of pinhole defects.

The growth modes are usually dependent on the electrode size. For large-size electrodes, the electrodeposition process is generally under kinetic control due to sufficient surface area and electrolyte exchange capacity, which means that the nucleation rate is the main determinant of the deposition rate. For small-size electrodes, like UMEs, with a relatively small surface area and limited electrolyte exchange capacity, the deposition process is more likely to be under diffusion control. The existing models are often unable to account for planar diffusion jumps of the small electrode due to the overlap of diffusion boundary layers. Therefore, new models need to be developed to better describe the variation in planar diffusion. In addition, electrodes with smaller surface areas may exhibit stronger edge effects. This review also examines the preparation of pinhole-free layers of different scales by electrodeposition, which is used in the fields of solar cells and tunnelling ultramicroelectrodes.

The composition, morphology and surface density of the substrate are key determinants of film growth. According to the previous statement, a lattice fit should be considered when selecting substrates to minimise lattice strain and interfacial defects. A lower surface density helps to reduce the diffusion of atomic exchanges between the substrate and the deposited material, reducing the formation of impurities and pinhole defects. The most common pinhole defects are open pinholes, closed pinholes, keyholes and pit holes, the formation of which is summed up as the shadowing effect, coalescence of point vacancies, cross-layer migration, the existence of internal stress and attached hydrogen bubbles in this review. Marked progress has been obtained during recent decades; however, there are still challenges that need to be solved as follows: (1) Progress in understanding the mechanism of defect generation and propagation is insufficient, and the application of multiphysics models and software of different scales is required. (2) The reproducibility and consistency of electrodeposition technologies must be improved for industrial batch production. (3) The current transient phenomena observed in the electrodeposition consisting of multi-step reactions are important and should be explained by considering the kinetics of charge/mass transfer and linear diffusion on planar electrodes. The “current-time” response of the multi-step reactions is mainly caused by the limiting step, which affects the gradual nucleation and growth process of the deposits. Consideration of the complex electrochemical behaviour within the metal or metal oxide deposits, rather than only focusing on the specific properties of the electrode surface, is highly necessary.

## Data Availability

Not applicable.
